# New cycloartane glycosides from the aerial part of *Thalictrum fortunei*

**DOI:** 10.1007/s11418-012-0675-6

**Published:** 2012-06-12

**Authors:** Xian-Tao Zhang, Lei Wang, Shu-Wei Ma, Qing-Wen Zhang, Yue Liu, Lei-Hong Zhang, Wen-Cai Ye

**Affiliations:** 1 Guangdong Research Institute of Traditional Chinese Medicine, Guangzhou, 510520 People’s Republic of China; 2 Institute of Traditional Chinese Medicine and Natural Products, College of Pharmacy, Jinan University, Guangzhou, 510632 People’s Republic of China; 3 Department of Pharmacy Engineering, Institute of Chemistry and Chemical Engineering, Qiqihar University, Qiqihar, 16100 People’s Republic of China; 4 Institute of Chinese Medical Sciences, University of Macau, Taipa, Macau, People’s Republic of China

**Keywords:** *Thalictrum fortunei*, Ranunculaceae, Cycloartane glycoside

## Abstract

Four new cycloartane glycosides, 3-*O*-*β*-d-xylopyranosyl-(1 → 6)-*β*-d-glucopyranosyl-(1 → 4)-*β*-d-fucopyranosyl (22*S*,24*Z*)-cycloart-24-en-3*β*,22,26-triol 26-*O*-(6-*O*-acetyl)-*β*-d-glucopyranoside (**1**), 3-*O*-*α*-l-arabinopyranosyl-(1 → 6)-*β*-d-glucopyranosyl-(1 → 4)-*β*-d-fucopyranosyl (22*S*,24*Z*)-cycloart-24-en-3*β*,22,26-triol 26-*O*-(6-*O*-acetyl)-*β*-d-glucopyranoside (**2**), 3-*O*-*β*-d-glucopyranosyl (24*S*)-cycloartane-3*β*,16*β*,24,25,30-pentaol 25-*O*-*β*-d-glucopyranosyl-(1 → 6)-*β*-d-glucopyranoside (**3**) and 3-*O*-*β*-d-glucopyranosyl (24*S*)-cycloartane-3*β*,16*β*,24,25,30-pentaol 25-*O*-*β*-d-glucopyranosyl-(1 → 4)-*β*-d-glucopyranoside (**4**), were isolated from the aerial parts of *Thalictrum fortunei*. Their structures were established on the basis of extensive NMR and HR-ESI-MS analyses, along with acid hydrolysis.

## Introduction

**Fig. 1:**
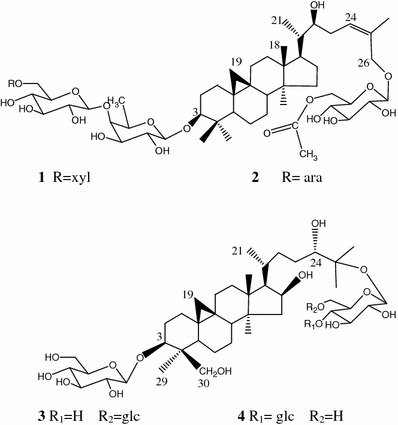
Chemical structures of **1**–**4**

The plant *Thalictrum* *fortunei* S. Moore (Ranunculaceae) is mainly distributed throughout southeastern China. The aerial part of this plant has been used in traditional Chinese medicine for the treatment of ophthalmia, dysentery and jaundice [1, 2]. In our previous study, several new cycloartane glycosides were isolated from this plant [3–5]. Further investigation on the aerial parts of the plant has led to the isolation of four new saponins (**1**–**4**) (Fig. 1), whose structures were determined with the aid of NMR, HR-ESI-MS and acid hydrolysis. Herein, we report the isolation and structural elucidation of these new compounds.

## Results and discussion

**Fig. 2:**
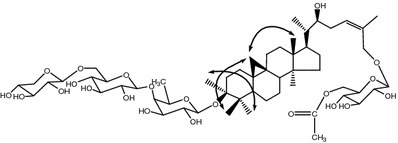
Key ROESY correlations of compound **1**

Compound **1** was obtained as a white powder. Positive results from both Liebermann–Burchard and Molisch reactions indicated that **1** was a saponin. The molecular formula of **1** was C_55_H_90_O_22_ as determined from the quasimolecular ion [M−H]^−^ at *m*/*z* 1101.5763 (calcd for C_55_H_89_O_22_: 1101.5846) in its HR-ESI mass spectrum. Acid hydrolysis of **1** afforded d-glucose, d-xylose and d-fucose, which were determined by gas chromatography analysis. The ^1^H NMR spectrum of **1** exhibited two doublet signals at *δ* 0.21 and 0.48 (each 1H, d, *J* = 3.8 Hz), which are characteristic for a cyclopropane moiety [6]. In addition, the signals for six tertiary methyls at *δ* 0.85, 1.00, 1.03, 1.28, 1.92 and 1.99 (each 3H, s), two secondary methyls at *δ* 1.16 (3H, d, *J* = 6.6 Hz) and 1.69 (3H, d, *J* = 6.1 Hz), as well as four anomeric protons at *δ* 4.66 (1H, d, *J* = 7.5 Hz), 4.85 (1H, d, *J* = 7.8 Hz), 4.95 (1H, d, *J* = 7.6 Hz) and 5.11(1H, d, *J* = 7.8 Hz) were observed. The ^13^C NMR and DEPT spectra displayed 55 carbon signals, including two olefinic carbons (*δ* 128.5 and 133.2) and four anomeric carbons (*δ* 106.9, 106.6, 106.0 and 102.8). With the aid of ^1^H–^1^H COSY, HSQC, HMBC and ROESY experiments (Fig. 2), all the ^1^H and ^13^C NMR signals of **1** were assigned as shown in Tables 1 and 2. A comparison of the NMR data of **1** with those of the known compound 3-*O*-*β*-d-glucopyranosyl-(1 → 4)-*β*-d-fucopyranosyl (22*S*,24*Z*)-cycloart-24-en-3*β*,22,26-triol 26-*O*-*β*-d-glucopyranoside revealed that their NMR signals were very similar, except for the appearance of the signals for additional acetyl and xylose units in **1**, indicating that the aglycone of **1** is (22*S*,24*Z*)-cycloart-24-en-3*β*,22,26-triol [3]. The linkage sequence and positions of sugar moieties were determined by an HMBC experiment. Hence, in the HMBC spectrum, correlations between H-1 (*δ* 4.95) of xylose and C-6 (*δ* 70.0) of glucose, between H-1 (*δ* 5.11) of glucose and C-4 (*δ* 83.0) of fucose, between H-1 (*δ* 4.66) of fucose and C-3 (*δ* 88.6) of aglycone, as well as between H-1′ (*δ* 4.85) of another glucose and C-26 (*δ* 67.4) of aglycone, as well as between H-6′ (*δ* 4.73) of the glucose and carbonyl (*δ* 170.9) of the acetyl group were clearly observed. Therefore, the structure of **1** was identified as 3-*O*-*β*-d-xylopyranosyl-(1 → 6)-*β*-d-glucopyranosyl-(1 → 4)-*β*-d-fucopyranosyl (22S,24Z)-cycloart-24-en-3*β*,22,26-triol 26-*O*-(6-*O*-acetyl)-*β*-d-glucopyranoside.

**Table 1:** ^13^C NMR data of **1**–**4** (pyridine-*d*_5_)

	**1**	**2**	**3**	**4**		**1**	**2**	**3**	**4**
1	32.2	31.1	32.0	32.0	4	71.5	70.6	71.7	71.8
2	30.0	28.9	30.2	29.9	5	77.4	76.1	78.4	78.2
3	88.6	87.4	89.3	89.3	6	70.0	68.7	62.7	62.9
4	41.3	40.2	45.0	45.0	Glc′ 1′	102.8	101.7	97.6	97.6
5	48.0	46.9	47.8	47.7	2′	75.3	74.1	75.4	75.4
6	21.2	20.0	21.9	21.8	3′	78.7	77.3	78.8	78.7
7	26.2	25.1	26.7	26.5	4′	71.4	70.3	71.6	82.7
8	47.7	46.5	48.8	48.4	5′	78.4	77.3	77.5	78.0
9	20.1	18.9	21.1	21.2	6′	64.8	63.7	70.2	62.7
10	26.4	25.2	25.6	25.5	Glc″ 1″			105.5	106.8
11	26.7	25.6	26.2	26.3	2″			75.2	75.5
12	33.4	32.3	33.2	33.0	3″			78.4	78.5
13	45.5	44.3	45.6	45.7	4″			71.8	71.6
14	49.0	48.0	46.8	46.9	5″			78.3	78.0
15	35.9	34.7	48.9	48.0	6″			62.7	62.9
16	28.0	26.9	71.6	71.7	Fuc 1	106.9	105.8		
17	49.1	48.0	57.6	57.7	2	73.5	72.4		
18	18.3	17.2	19.6	19.6	3	75.7	74.6		
19	29.7	28.6	29.9	30.3	4	83.0	82.0		
20	41.7	40.5	29.4	29.5	5	70.4	69.3		
21	12.1	11.0	18.2	17.6	6	17.9	16.8		
22	73.0	71.8	33.8	34.0	Xyl 1	106.0			
23	35.0	33.8	28.4	28.8	2	74.9			
24	128.5	127.5	74.8	74.9	3	78.1			
25	133.2	132	80.5	80.7	4	71.2			
26	67.4	66.3	22.9	22.8	5	67.0			
27	22.2	21.0	22.5	22.4	Ara 1		104.4		
28	19.6	18.5	20.3	20.4	2		71.2		
29	25.8	24.7	21.2	21.0	3		73.1		
30	15.4	14.3	63.3	63.3	4		67.9		
Glc 1	106.6	105.5	106.1	106.2	5		65.3		
2	75.8	74.6	75.5	75.6	OAc	170.9	167.9		
3	78.6	77.5	78.7	78.5		21.0	20.8		

*Glc* *β*-d-glucopyranose, *Fuc* *β*-d-fucopyranose, *Xyl* *β*-d-xylopyranose, *Ara* *α*-l-arabinopyranose

**Table 2:** ^1^H NMR data of saccharide moieties for **1**–**4** (pyridine-*d*_5_, *δ*, *J* in Hz)

	**1**	**2**		**3**	**4**
Glc 1	5.11 (d, 7.8)	5.09 (d, 7.7)	Glc 1	4.99 (d, 7.8)	5.02 (d, 7.8)
2	3.90 (dd, 7.8, 9.0)	3.98 (dd, 7.7, 9.2)	2	3.94 (dd, 7.8, 9.0)	3.90 (dd, 7.8, 9.1)
3	4.10	4.12	3	4.17	4.10
4	4.15	4.02	4	4.19	4.16
5	3.96	3.94	5	3.85	3.98
6a	4.72 (br d, 11.4)	4.72 (br d, 9.6)	6a	4.44 (dd, 11.8, 2.5)	4.49 (dd, 11.8, 2.5)
6b	4.26 (dd, 11.4, 4.3)	4.21 (dd, 9.6, 4.5)	6b	4.28 (dd, 11.8, 5.3)	4.32 (dd, 11.8, 5.3)
Glc′ 1′	4.85 (d, 7.8)	4.80 (d, 7.8)	Glc′ 1′	5.09 (d, 7.8)	5.17 (d, 7.8)
2′	4.00 (dd, 7.8, 9.2)	3.98 (dd, 7.8, 9.0)	2′	3.93 (dd, 7.8, 9.0)	3.88 (dd, 7.8, 9.2)
3′	4.20	4.12	3′	4.19	4.16
4′	4.21	4.10	4′	4.14	4.36
5′	4.04	4.03	5′	4.11	3.83
6a′	4.92 (br d, 11.9)	4.92 (br d, 11.9)	6a′	4.82 (br d, 11.8)	4.38 (br d, 11.8)
6b′	4.73 (dd, 11.9, 4.3)	4.74 (dd, 11.9, 4.3)	6b′	4.29 (dd, 11.8, 2.5)	4.25 (dd, 11.8, 5.2)
Fuc 1	4.66 (d, 7.5)	4.66 (d, 7.5)	Glc″ 1″	5.16 (d, 7.7)	4.66 (d, 7.6)
2	4.27 (dd, 7.5, 8.0)	4.27 (dd, 7.5, 8.0)	2″	4.03 (dd, 7.7, 9.0)	3.83 (dd, 7.6, 9.0)
3	3.98	3.98	3″	3.94	4.10
4	4.01	3.99	4″	4.05	4.13
5	3.71 (dq, 13.2, 6.4)	3.72 (dq, 13.3, 6.5)	5″	3.85	3.83
6	1.69 (d, 6.4)	1.69 (d, 6.5)	6a″	4.51 (br d, 11.8)	4.38 (br d, 11.8)
Xyl/Ara	4.95 (d, 7.6)	4.89 (d, 6.5)	6b″	4.34 (dd, 11.8, 2.5)	4.25 (dd, 11.8, 2.5)
2	4.00	4.41 (dd, 6.5, 6.8)			
3	3.90	4.09			
4	4.10	4.22			
5a	4.27 (d, 11.3)	4.18			
5b	3.63 (dd, 11.3, 10.0)	3.68			
OAc	1.99 (s)	1.99 (s)			

*Glc* *β*-d-glucopyranose, *Fuc* *β*-d-fucopyranose, *Xyl* *β*-d-xylopyranose, *Ara* *α*-l-arabinopyranose

Compound **2** was obtained as a white powder. Positive results from both Liebermann–Burchard and Molisch reactions indicated that **2** was a saponin. The molecular formula of **2** was determined to be C_55_H_90_O_22_ by HR-ESI-MS (*m*/*z* 1101.5763 [M−H]^−^; calcd for C_55_H_89_O_22_: 1101.5846). Acid hydrolysis of **2** afforded d-glucose, l-arabinose and d-fucose. The ^1^H NMR spectrum of **2** showed signals for a cyclopropane unit at *δ* 0.20 and 0.45 (each 1H, d, *J* = 3.8 Hz), six tertiary methyls at *δ* 0.85, 0.99, 1.03, 1.28, 1.91 and 1.99 (each 3H, s), two secondary methyls at *δ* 1.16 (3H, d, *J* = 6.6 Hz) and 1.69 (3H, d, *J* = 7.3 Hz), as well as four anomeric protons at *δ* 4.66 (1H, d, *J* = 7.5 Hz), 4.80 (1H, d, *J* = 7.8 Hz), 4.89 (1H, d, *J* = 6.5 Hz) and 5.09 (1H, d, *J* = 7.7 Hz), indicating that **2** was also a cycloartane glycoside. Comparison of the ^13^C NMR data of **2** with those of **1** revealed that they possessed the same aglycone, but the signals of the xylose in **1** were replaced by those of an arabinose (*δ* 104.4, 73.1, 71.2, 67.9, 65.3) [3, 7] (Table 1). The linkage sequence and positions of sugar moieties were confirmed by the HMBC correlations between H-1 (*δ* 4.89) of arabinose and C-6 (*δ* 68.7) of glucose, between H-1 (*δ* 5.09) of glucose and C-4 (*δ* 82.0) of fucose, between H-1 (*δ* 4.66) of fucose and C-3 (*δ* 87.4) of aglycone, as well as between H-1′ (*δ* 4.80) of another glucose and C-26 (*δ* 66.3) of aglycone. These findings led to the assignment of **2** as 3-*O*-*α*-l-arabinopyranosyl-(1 → 6)-*β*-d-glucopyranosyl-(1 → 4)-*β*-d-fucopyranosyl (22*S*,24*Z*)-cycloart-24-en-3*β*,-22,-26-triol 26-*O*-(6-*O*-acetyl)-*β*-d-glucopyranoside.

Compound **3** was obtained as a white powder. Positive results from both Liebermann–Burchard and Molisch reactions indicated the compound was a saponin. The molecular formula was determined to be C_48_H_82_O_20_ by HR-ESI-MS (*m*/*z* 977.5340 [M−H]^−^; calcd for C_48_H_81_O_20_: 977.5321). Acid hydrolysis of **3** afforded d-glucose. The ^1^H NMR spectrum showed two doublet signals at *δ* 0.23 and 0.45 (each 1H, d, *J* = 3.8 Hz), which is characteristic for a cyclopropane moiety. Furthermore, signals for three anomeric protons at *δ* 4.99 (1H, d, *J* = 7.8 Hz), 5.09 (1H, d, *J* = 7.8 Hz) and 5.16 (1H, d, *J* = 7.7 Hz), five tertiary methyls at *δ* 0.81, 1.34, 1.43, 1.49 and 1.53 (each 3H, s), and a secondary methyl at *δ* 1.06 (3H, d, *J* = 6.6 Hz) were observed. Comparison of the ^13^C NMR spectrum of **3** with those of the known compound 3-*O*-*β*-d-glucopyranosyl (24*S*)-cycloartane-3*β*,16*β*,24,25,30-pentaol 25-*O*-*β*-d-glucopyranoside [4] indicated that the aglycone of **3** was 24*S*-cycloartane-3*β*,16*β*,24,25,30-pentaol (cyclofoetigenin B, a known compound) [8, 9], which was further confirmed by the ^1^H–^1^H COSY, HSQC, HMBC and ROESY data. The sequence and linkage positions of the sugar moieties were determined by an HMBC experiment. In the HMBC spectrum, correlations between H-1(*δ* 4.99) of glucose and C-3 (*δ* 89.3) of aglycone, between H-1″ (*δ* 5.16) of the outer glucose and C-6′ (*δ* 70.2) of the inner glucose, as well as between H-1′ (*δ* 5.09) of the inner glucose and C-25 (*δ* 80.5) were observed. On the basis of the above evidence, the structure of **3** was established as 3-*O*-*β*-d-glucopyranosyl (24*S*)-cycloartane-3*β*,16*β*,24,25,30-pentaol 25-*O*-*β*-d-glucopyranosyl-(1 → 6)-*β*-d-glucopyranoside.

Compound **4** was obtained as a white powder. Positive results from both Liebermann–Burchard and Molisch reactions indicated that **4** was a saponin. The molecular formula was determined to be C_48_H_82_O_20_ by HR-ESI-MS (*m*/*z* 977.5348 [M−H]^−^; calcd for C_48_H_81_O_20_: 977.5321). Acid hydrolysis of **4** afforded d-glucose. The ^1^H-NMR spectrum also showed two doublet signals at *δ* 0.16 and 0.33 (each 1H, d, *J* = 3.8 Hz), which is characteristic for a cyclopropane unit. The ^1^H and ^13^C NMR data of **4** were similar to those of the reported compound 3-*O*-*β*-d-glucopyranosyl (24*S*)-24-*O*-acetyl-cycloartane-3*β*,16*β*,24,25,30-pentaol 25-*O*-*β*-d-glucopyranosyl-(1 → 4)-*β*-d-glucopyranoside [4], except that the signals due to the acetyl moiety were absent in **4**. The ^1^H and ^13^C NMR data (Tables 1, 2) were assigned by a combination of ^1^H–H COSY, HSQC, HMBC and ROESY experiments. The sequence and linkage positions of the sugar moieties were confirmed by an HMBC experiment. Hence, in the HMBC spectrum, the correlations between H-1 (*δ* 5.02) of glucose and C-3 (*δ* 89.3) of aglycone, between H-1″ (*δ* 4.66) of the outer glucose and C-4′ (*δ* 82.7) of the inner glucose, as well as between H-1′ (*δ* 5.17) of the inner glucose and C-25 (*δ* 80.7) of aglycone were observed. On the basis of the above evidence, the structure of **4** was established as 3-*O*-*β*-d-glucopyranosyl (24*S*)-cycloartane-3*β*,16*β*,24,25,30-pentaol 25-*O*-*β*-d-glucopyranosyl-(1 → 4)-*β*-d-glucopyranoside.

## Experimental

### General experimental procedures

Melting points were determined on an X-4 apparatus (Ningbo Hinotek Technology Ltd., Ningbo, China) and are uncorrected. Optical rotations were obtained using a Perkin-Elmer 241 polarimeter (Perkin-Elmer Ltd., Norwalk, USA). IR spectra were measured on a Nicolet Impact 410 FT-IR instrument (Nicolet Instrument Ltd., Madison, USA). UV spectra were recorded on a Shimadzu UV-2501 spectrophotometer (Shimadzu Ltd., Kyoto, Japan). The ^1^H and ^13^C NMR spectra were obtained on a Bruker AV500 Avance spectrometer (^1^H, 500 MHz; ^13^C, 125 MHz; Bruker Ltd., Karlsruhe, Germany) and chemical shifts are given in *δ* (ppm) with TMS as a reference. HR-ESI-MS data were obtained on an Applied Biosystems Mariner 5140 spectrometer (Life Technologies Ltd., New York, USA). Column chromatography (CC) was performed on silica gel (Qingdao Marine Chemical Ltd., Qingdao, China) and ODS (Merck Ltd., Darmstadt, Germany). Thin-layer chromatography was performed on precoated silica gel GF_254_ plates (Qingdao Marine Chemical Ltd., Qingdao, China). Preparative HPLC was carried out using a Zobax XDB-18 column (10 mm i.d. × 15 cm, Agilent Technologies Ltd., Wilmington DE, USA). GC experiments were carried out on an HP-1 TCD instrument (Hewlett-Packard Ltd., Palo Alto, USA) using an HP-Chiral column (30 m × 0.25 mm × 1.0 μm, 20 % permethylated *β*-cyclodextrin; Agilent Technologies Ltd., Wilmington DE, USA). All chemical reagents were purchased from Nanjing Reagent Co., Ltd. (Nanjing, China).

### Plant material

The aerial parts of *Thalictrum fortunei* were collected in April of 2004 in Wuhu city, Anhui province of China, and were authenticated by Prof. Min-Jian Qin (China Pharmaceutical University). A voucher specimen (No. 040192) was deposited in the Herbarium of China Pharmaceutical University, Nanjing, People’s Republic of China.

### Extraction and isolation

The dried aerial parts (4.8 kg) of *T. fortunei* were extracted with 95 % EtOH (3 × 20 L) under reflux. The EtOH extract was suspended in water and then successively extracted with petroleum ether, EtOAc and *n*-BuOH. The *n*-BuOH solution was concentrated to give a residue (207 g), which was separated by silica gel CC using CHCl_3_/MeOH (1:0 → 1:1, v/v) as eluent, affording five fractions (frs. 1–5). Fr 5 (889 mg) was further purified by ODS CC eluted with MeOH/H_2_O (70:30, v/v) and preparative HPLC using CH_3_CN/H_2_O (34:66, v/v) as solvent to yield compound **1** (25 mg,* t*_R_ 7.3 min), **2** (30 mg,* t*_R_ 9.8 min), **3** (17 mg,* t*_R_ 17.9 min) and **4** (22 mg,* t*_R_ 22.4 min).

### 3-*O*-*β*-d-Xylopyranosyl-(1 → 6)-*β*-d-glucopyranosyl-(1 → 4)-*β*-d-fucopyranosyl (22*S*,24*Z*)-cycloart-24-en-3*β*,22,26-triol 26-*O*-(6-*O*-acetyl)-*β*-d-glucopyranoside (**1**)

White powder, [*α*]$\mathop D\limits^{25}$ −25.8 (*c* 0.20, MeOH); mp: 232–233 °C; IR (KBr) *v*_max_: 3465, 3414, 1603, 1387, 1360, 1120, 771, 625, 473 cm^−1^; HR-ESI-MS *m*/*z*: 1101.5763 [M−H]^−^ (calcd for C_55_H_89_O_22_: 1101.5846); ^1^H NMR (pyridine-*d*_5_): *δ* 0.48 (1H, d, *J* = 3.8 Hz, H-19a), 0.21 (1H, d, *J* = 3.8 Hz, H-19b), 0.85 (3H, s, Me-28), 1.00 (3H, s, Me-30), 1.03 (3H, s, Me-18), 1.16 (3H, d, *J* = 6.6 Hz, Me-21), 1.28 (3H, s, Me-29), 1.92 (3H, s, Me-27), 1.99 ((3H, s, OAc), 3.40 (1H, dd, *J* = 11.7, 4.3 Hz, H-3), 4.49, 4.70 (each 1H, ABq, *J* = 12.0 Hz, H-26a, -26b), 5.77 (1H, t, *J* = 7.2 Hz, H-24); ^1^H NMR data of the saccharide residues, see Table 2; ^13^C NMR data, see Table 1.

### 3-*O*-*α*-l-Arabinopyranosyl-(1 → 6)-*β*-d-glucopyranosyl-(1 → 4)-*β*-d-fucopyranosyl (22*S*,24*Z*)-cycloart-24-en-3*β*,22,26-triol 26-*O*-(6-*O*-acetyl)-*β*-d-glucopyranoside (**2**)

White powder, [*α*]$\mathop D\limits^{25}$ −6.9 (*c* 0.15, MeOH); mp: 223–224 °C; IR (KBr) *v*_max_: 3427, 1604, 1381, 1118, 773, 624, 460 cm^−1^; HR-ESI-MS *m*/*z*: 1101.5763 [M−H]^−^ (calcd for C_55_H_89_O_22_: 1101.5846); ^1^H NMR (pyridine-*d*_5_): *δ* 0.45 (1H, d, *J* = 3.8 Hz, H-19a), 0.20 (1H, d, *J* = 3.8 Hz, H-19b), 0.85 (3H, s, Me-28), 0.99 (3H, s, Me-30), 1.03 (3H, s, Me-18), 1.16 (3H, d, *J* = 6.6 Hz, Me-21), 1.28 (3H, s, Me-29), 1.91 (3H, s, Me-27), 1.99 ((3H, s, OAc), 3.41 (1H, dd, *J* = 11.9, 4.1 Hz, H-3), 4.47, 4.70 (each 1H, ABq, *J* = 12.0 Hz, H-26a, -26b), 5.77 (1H, t, *J* = 7.2 Hz, H-24); ^1^H NMR data of the saccharide residues, see Table 2; ^13^C NMR data, see Table 1.

### 3-*O*-*β*-d-Glucopyranosyl (24*S*)-cycloartane-3*β*,16*β*,24,25,30-pentaol 25-*O*-*β*-d-glucopyranosyl-(1 → 6)-*β*-d-glucopyranoside (**3**)

White powder, [*α*]$\mathop D\limits^{25}$ +0.1 (*c* 1.01, MeOH); mp: 202–204 °C; IR (KBr) *v*_max_: 3320, 2924, 2854, 1462, 1377, 1099, 1025 cm^−1^; HR-ESI-MS *m*/*z*: 977.5340 [M−H]^−^ (calcd for C_48_H_81_O_20_: 977.5321); ^1^H NMR (pyridine-*d*_5_): *δ* 0.23 (1H, d, *J* = 3.8 Hz, H-19a), 0.45 (1H, d, *J* = 3.8 Hz, H-19b), 0.81 (3H, s, Me-28), 1.06 (3H, d, *J* = 6.6 Hz, Me-21), 1.34 (3H, s, Me-18), 1.43 (3H, s, Me-27), 1.49 (3H, s, Me-26), 1.53 (3H, s, Me-29), 3.83 (1H, dd, *J* = 11.9, 4.3 Hz, H-3), 4.01 (1H, dd, *J* = 10.0, 1.7 Hz, H-24); ^1^H NMR data of the saccharide residues, see Table 2; ^13^C NMR data, see Table 1.

### 3-*O*-*β*-d-Glucopyranosyl (24*S*)-cycloartane-3*β*,16*β*,24,25,30-pentaol 25-*O*-*β*-d-glucopyranosyl-(1 → 4)-*β*-d-glucopyranoside (**4**)

White powder, [*α*]$\mathop D\limits^{25}$ +4.8 (*c* 1.00, MeOH); mp: 176–178 °C; IR (KBr) *v*_max_: 3289, 2937, 1590, 1454, 1383, 1041 cm^−1^; HR-ESI-MS *m*/*z*: 977.5348 [M−H]^−^ (calcd for C_48_H_81_O_20_: 977.5321); ^1^H NMR (pyridine-*d*_5_): *δ* 0.33 (1H, d, *J* = 3.8 Hz, H-19a), 0.16 (1H, d, *J* = 3.8 Hz, H-19b), 0.80 (3H, s, Me-28), 1.00 (3H, d, *J* = 6.6 Hz, Me-21), 1.20 (3H, s, Me-18), 1.51 (3H, s, Me-27), 1.58 (3H, s, Me-26), 1.62 (3H, s, Me-29), 3.77 (1H, dd, *J* = 11.9, 4.3 Hz, H-3), 4.02 (1H, dd, *J* = 10.0, 1.8 Hz, H-24); ^1^H NMR data of the saccharide residues, see Table 2; ^13^C NMR data, see Table 1.

### Acid hydrolysis

The solution of each compound (**1**–**4**, each 5 mg) in 30 mL of 1 M HCl (MeOH/H_2_O, 1:1, v/v) was heated under reflux for 3 h. After removal of the solvent, the residue was partitioned between CHCl_3_ and H_2_O. The aqueous layer was neutralized with Dowex (HCO_3_^−^) and filtered. The filtrate was concentrated to 2 mL and then treated with NaBH_4_ (20 mg) at room temperature for 3 h. Excess NaBH_4_ was removed with 30 % AcOH. After evaporation at 60 °C and washing with 0.1 % HCl/MeOH, the mixture was dried at 105 °C for 15 min. After the addition of pyridine (0.5 mL) and Ac_2_O (0.5 mL), the mixture was incubated in a water bath at 100 °C for 1 h, and then partitioned between CHCl_3_ and H_2_O. The CHCl_3_ layer was concentrated for GC analysis (front inlet 250 °C, column temp. 80 °C → 230 °C, 5 °C/min)[10]. The peaks of each monosaccharide derivative were observed at *t*_R_ (min): **1**: d-glucose 33.079, d-fucose 27.829, d-xylose 28.810; **2**: d-glucose 33.067, l-arabinose 28.293, d-fucose 27.835; **3**: d-glucose 33.064; **4**: d-glucose 33.071 (reference d-fucose 27.835, l-fucose 29.360, d-xylose 28.789, l-xylose 30.695, d-glucose 33.077, l-glucose 34.460, d-arabinose 29.862, l-arabinose 28.288).
